# Granular cell tumor of the larynx in children: a case report

**DOI:** 10.1016/S1808-8694(15)31391-4

**Published:** 2015-10-17

**Authors:** Wanessa Alessandra Ruiz Scala, Alessandro Murano Ferre Fernandes, André de C. Duprat, Henrique O. Olival Costa

**Affiliations:** 1 MSc student in Otorhinolaryngology at Santa Casa de SP; 2 MSc in Medicine at Faculdade de Ciências Médicas da Santa Casa de São Paulo. PhD student at Faculdade de Ciências Médicas da Santa Casa de São Paulo. Instructor Professor at the Department of Otorhinolaryngology at Santa Casa de Misericórdia de São Paulo; 3 MSc in Medicine at Faculdade de Ciências Médicas da Santa Casa de São Paulo. Instructor Professor at the Department of Otorhinolaryngology at Santa Casa de Misericórdia de São Paulo; 4 MSc in Medicine at Faculdade de Ciências Médicas da Santa Casa de São Paulo. Assistant Professor at the Department of Otorhinolaryngology at Santa Casa de Misericórdia de São Paulo. Department of Otorhinolaryngology of the Santa Casa de Misericórdia de São Paulo R. Cesário Mota Jr, 112 - Santa Cecília - São Paulo Phone # +55 11 3224-0122 / 3226-7235

**Keywords:** larynx, granular cells tumor

## Abstract

The granular cell tumor (GCT) is an uncommon neoplasm, with slow progression, usually benign, that can be found in any organ. The most common region for GCT involvement is in the head and neck. Laryngeal involvement is uncommon and accounts for 6 to 10% of all cases reported. Among the major theories of origin and based on the strongest evidence, the most accepted one is that the tumor stems from neuronal tissue. The GCT has a higher incidence in African-descendent patients, and most commonly in their 4th and 6th decades of life. The posterior larynx is the most common laryngeal site. Pediatric laryngeal GCT is rare, anterior subglottis involvement has been described and extensive glottic involvement may occur. Affected patients typically present with hoarseness, dysphagia, cough, haemoptysis, stridor and pain. The GCT presents as a small, firm nodule, sessile or polypoid, with intact mucosa, well outlined but not encapsulated. Cytoplasm granules are typically seen under light microscopy, and the cells are positive for S100 immunoperoxidase and neuron-specific enolase. Treatment of laryngeal GCT is based on surgical excision. This paper describes a pediatric patient with GCT and its clinical course before and after surgical treatment, stressing the importance of GCT diagnosis in the pediatric population. We review clinical course, pathology characteristics and treatment.

## INTRODUCTION

Granular cell tumors are uncommon, mostly benign neoplasms that may involve any of the bodily organs. GCTs were first described by Abrikossoff in 1926 and categorized as myoblastomas, as they were supposedly originated in muscle cells. These tumors were given names other than myoblastoma, such as myoblastic tumor, granular cell myoblastoma, uniform myoblastoma, embryonal rhabdomyoblastoma, granular cell neuroma, granular cell schwannoma, and congenital epulis. However, granular cell tumor (GCT) is the nomenclature adopted by the World Health Organization^1^ (WHO).

GCTs are more often found in male adults above the age of 40, with head and neck as the most frequently involved sites. Manifestations in otorhinolaryngological sites are rare, and tumors are more often found in the tongue and larynx^2^, ^4^, ^6^. This neoplasm is rarely found in children, and only 20 cases have been reported in the literature^12^, ^13^. Airway involvement leads to respiratory disorders such as dyspnea and stridor, while laryngeal tumors are identified accidentally during laryngoscopy.^4^

This paper describes the case of an 11-year-old patient diagnosed with laryngeal granular cell tumor and the patient’s clinical evolution after tumor surgical removal. A literature review was carried out to analyze the clinical and pathological characteristics of the tumor, as well as the current treatment options. The rarity of the case and its clinical presentation are relevant in producing the differential diagnosis against other pediatric laryngeal diseases.

## LITERATURE REVIEW

Granular cell tumors were first described by Abrikossoff in 1926 and were called myoblastomas, as they were believed to originate in muscle cells. GCTs were also known as Abrikossoff’s tumor^3^. In 1935, Feyrter postulated that these tumors had a neural origin, thus calling them granular cell neuromas. His theory was confirmed by Fust and Custer in 1948, who proposed a new name - granular cell neurofibroma. In 1962, Fisher and Wechsler conducted ultrastructural and immunohistochemistry studies to find that Schwann’s cells were the most likely origin of these tumors, and thus named them granular cell schwannomas^1^.

In spite of the arguments around the origin of GCTs, the apparent connection with Schwann’s cells is based on solid findings: ultrastructural similarity between Schwann’s and granular cells; similarity between the granules in granular cells and altered myelin; concentric arrangement of granular cells around nerve ends; presence of lipoproteins and sphingomyelin in granular cells indicating that the granules in granular cells are made of myelin or the product of its degradation; and positivity for protein S100, enolase, and myelinic proteins PO and P2 by immunoperoxidase techniques.

Some authors found that GCTs are more commonly found in males and people of African descent1, between the fourth and fifth decades of life. Conde-Jahn et al. found a slightly higher prevalence in females^6^, while Lazar et al. described prevalence rates among women twice as high as those found among men8. Laryngeal involvement is more found in slightly younger men, with median age of 36 years^8^, ^10^. Only a handful of pediatric cases have been described in the literature.

Granular cell tumors are neoplasms of rare occurrence that may involve any of the bodily organs, occasionally appearing in two or more sites. About 50% of the tumors are found in the head and neck area^4^, ^5^, involving skin, subcutaneous tissue, and mucosa. The anterior portion of the tongue is the most compromised site, followed by larynx and tonsillar pillars^4^, ^6^, ^9^. GCT is a multicentric disease in 10% of the patients, with higher incidence rates when tumors are found in the airways^8^. Lower airways are involved in 15% of the cases^6^.

Laryngeal involvement is uncommon, and accounts for 6% to 10% of the GCT cases^2^, ^6^, ^8^. Despite the preferential involvement of the posterior larynx^3^, tumors may also be found in the anterior larynx, vocal folds, and arytenoids^6^. Anterior subglottic neoplasms and tumors extending towards the glottis are the most commonly described lesions in children. According to Conley et al., half of the pediatric patients have subglottic tumors, 62% of which located anteriorly^7^.

GCTs grow slowly, and evolve on average for 6 to 7 months before patients seek medical advice1. Hoarseness is the main symptom and is reported by over 90% of the patients. Dysphagia, pain, coughing, hemoptysis, and stridor may also be present and are related to the location and size of the tumor. Dyspnea may be the initial symptom in patients with subglottic tumor. Some patients experience symptoms similar to those seen in asthma, while others have silent tumors that are only found accidentally during laryngoscopic examination^4^, ^6^, ^7^, ^8^.

On the laryngoscope, GCTs appear to be firm, fixed or pedicled, non-ulcerated lumps, ranging from yellowish to grey-pinkish, usually with well-defined borders. They are not encapsulated and may frequently have infiltrative borders that simulate invasion^3^. Their size usually ranges between 0.3 and 3 cm^1^.

GCT diagnosis is done through pathology tests. The tumor is characterized by the presence of a large amount of dense cytoplasmatic lysosomes in different fragmentation stages, giving it a granular aspect under microscopy. The disease manifests itself in the form of subdermal or submucosal tumors with cells arranged in diffuse masses and strings. GCTs are characterized for not being encapsulated and for having imprecise borders. They may also invade and infiltrate adjacent tissues. The tumors are formed by large fusiform or polygonal cells with marked cell membrane and abundant pale cytoplasm, filled with eosinophilic granules. The most characteristic trait of granular cells is the membrane-contained cytoplasmatic granulation with microvesicles, increased density areas, microtubules, and myelinic formations. Cell nuclei are small, round to oval, located centrally, and some cells may have more than one. The pale, characteristic granules inside the cells are PAS (periodic acid-Schiff) positive and diastase-resistant, and may be sudanophilic, especially when stained using Sudan Black^3^, ^8^, ^10^. There is no peripheral inflammatory response.

The epithelium enveloping the tumor may present secondary pseudo-epitheliomatous epithelial hyperplasia in 50–65% of the cases, thus increasing diagnostic difficulty and leading to confusion in relation to epidermoid carcinoma^7^, ^8^, ^10^. In immunohistochemistry granular cell tumors are positive for protein S100 and neuron-specific enolase.

Congenital GCT, also known as congenital epulis, is in fact a variation of GCT with many similarities under the microscope but with evolutional, immunohistochemical, and ultrastructural differences.

The indicated treatment for laryngeal GCT is surgical removal. In the literature there is only one report of a case - an 11-year-old girl with Ewing’s sarcoma - treated with chemotherapy, with full remission of the tumor^4^, ^12^. Many are the surgical approaches to treat this condition, ranging from endoscopic surgery to laryngofissure and laryngectomy. Resection margins must be ample due to the tumor infiltration. Relapsing GCT is rare^3^, ^7^. Tumor relapse after surgical removal occurs in about 8% of the cases. When surgical margins are positive this rate increases to 21–50%, and 16% evolve to multiple tumors. Relapsing tumor surgical removal is usually a curative procedure.^4^

GCTs are almost always benign, but malignant manifestations are found in 1–3% of the patients. They are preferentially located in the skin and subcutaneous region and involve regional lymph nodes, although distal metastasis is uncommon. Malignancy is suspected from a series of factors1:

1. Cases of tumors macroscopically similar to benign GCTs, however quickly relapsing locally after surgical removal;
2. Tumors above 4 cm;
3. Tumor evolving slowly that suddenly begin to grow quickly;
4. Presence of distal metastasis;
5. Presence of atypia and pleomorphism, although not always present in malignant GCT.

Gamboa (1955) and Bastsaki and Manning (1995) reported GCT cases with malignant clinical and histological characteristics, with larger tumors (averaging 9 cm in diameter) in relation to benign GCTs. The name Atypical GCT was proposed for cases in which malignant histological traits and clinical aggressiveness are present, even without sings of metastasis^11^.

Malignant GCT has a poor prognosis, as patients die within 2–5 years after diagnosis4. Differential diagnosis is done against rhabdomyosarcoma, paraganglioma, oncocytic tumor, melanoma, and soft tissue alveolar sarcoma^11^.

## CLINICAL CASE PRESENTATION

J.N.C., 11, female, came to the ENT Service at Santa Casa de Misericórdia de São Paulo in December of 2002, complaining of constant hoarseness that had been affecting her for the past 3 years since she had an adenotonsillectomy. The condition progressed slowly and she got aphonic during colds. The patient also complained of dry cough and secretion, but referred no respiratory, gastric, or dysphagia symptoms. In May of 2002, the patient underwent a nasal fibroscopy at another service, and increased volume was observed in the middle and posterior thirds of her left vocal folds, without however limiting mobility. A biopsy was done, and two white fragments of fibroelastic consistence were removed. Pathology tests showed the presence of granular cell proliferation, with round non-atypical cells. Immunohistochemistry tests using immune-enzymatic techniques and the streptavidin-peroxidase method with monoclonal antibody analysis were done. The analyzed antigens were protein S100, enolase, Ki67, actin, desmin, and myoglobin; only the first two were positive.

**Figure 1:**
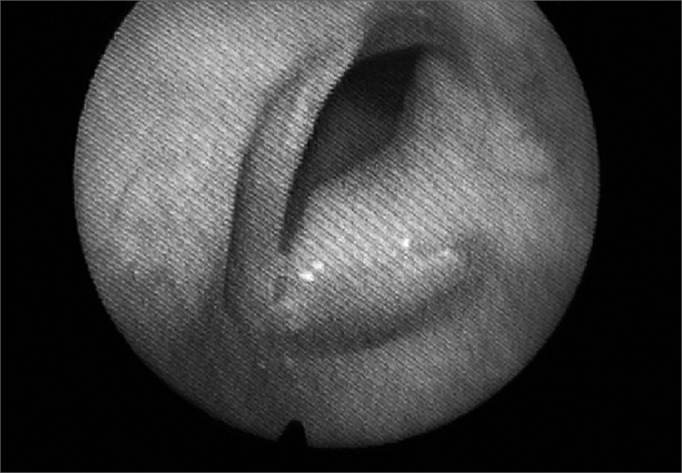
Laryngoscopy showing smooth-surface tumor involving the entire left vocal fold.

The patient was then assessed at our service by the laryngology department. Vocal quality alteration was found, as her voice was rough and blowy, with high pitch, limited modulation, reduced intensity, and shortened maximum phonation time. She underwent another nasal fibroscopy and a bulging left vocal fold all the way to the anterior commissure was seen, consequently to a glottic mass extending towards the subglottis. Its surface was smooth, and the color was similar to that of the rest of the vocal fold. (Fig. 1)

Another biopsy was done on the tumor through immunohistochemistry using the streptavidin-ABComplex method after heat antigenic recovery. Analysis was done for antigens protein S100 (strongly positive), actin (negative), and desmin (negative), suggestive of granular cell tumor.

MRI scans showed a thickened left vocal fold with paramagnetic contrast uptake, with a small projection into the glottic lumen that extended towards the subglottic region. Neck level II lymph nodes of approximately 1.0 cm were found to the left of the jugular-carotid chain.

The patient underwent a frontal-left laryngectomy and the tumor was fully removed. Frozen section biopsy was done during the procedure, and the margins were free of tumor. Macroscopic analysis of the surgical specimen showed the tumor had a cartilaginous, soft-tissue consistence, appeared to be firm, and homogeneously yellowish. Microscopically we observed a proliferation of round and polyhedral cells, with finely granular cytoplasm and dense, intensely stained nuclei, irregularly clustered and surrounded by bundles of collagen fiber. Specimen pathology tests showed fibroblastic proliferation with mucosal reactive vascular neoformation, confirming the diagnosis of laryngeal granular cell tumor (Fig. 2A and 2B).

**Figure 2A:**
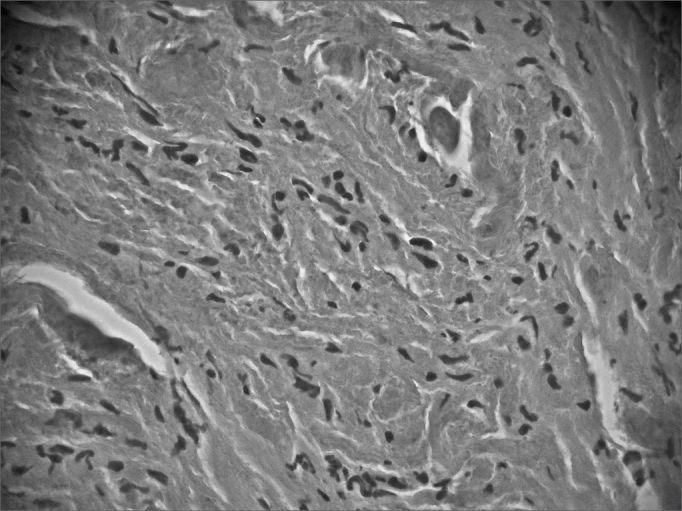
HE: histology of larger eosinophilic tumor cells with cytoplasmatic granulation (magnification 40 X 0.65); non-manipulated digitally captured image).

**Figure 2B:**
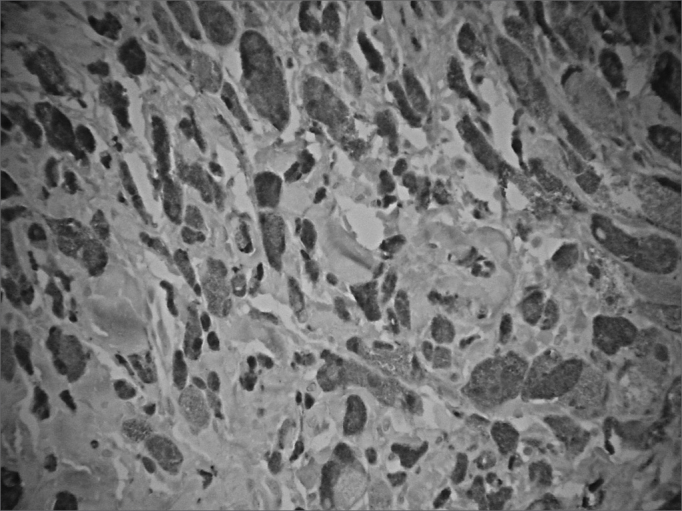
Immunohistochemical expression of protein S100-positive tumor cells.

After surgery the patient evolved satisfactorily, without dyspnea or pain. She is currently in her first year of post-op without signs of tumor relapse. The patient is having speech therapy sessions to improve her vocal patterns as a result of the phonation impacts introduced by the surgical procedure.

## DISCUSSION

Granular cell tumors are more frequently found in adults, with rare occurrences in the pediatric population. Laryngeal neoplasms are very rare in children, and usually include benign tumors as they account for 98% of the cases^13^. These tumors are accompanied by dyspnea, stridor, and upper airway obstruction due to airway lumen reduction. The table below shows the main pediatric laryngeal tumors (Table 1).

**Table 1:** Main pediatric laryngeal tumors

Benign	Malignant
Epithelial Squamous papilloma Mixed tumor Adenoma	Epithelial Squamous cell carcinoma Mixed tumor Adenocarcinoma Adenoid cystic carcinoma
Neurogenic Neurofibroma Granular cell tumor Neurilemmoma	Neurogenic Neurofibrosarcoma
Connective tissue Hemangioma Lipoma Rhabdomyoma Chondroma Fibroma	Connective tissue Fibrosarcoma Rhabdomyosarcoma Chondrosarcoma Angiosarcoma
Miscellaneous	Miscellaneous
Lymphangioma	Metastatic tumors
	Hematopoietic Lymphosarcoma Acute leukemia

This study presents the case of an 11-year-old patient with progressive hoarseness without history of dyspnea or dysphonia. Based on voice perceptual characteristics, the diagnostic possibilities revolve around vocal fold structural alterations - mainly nodules and epithelial cysts - but the patient complained of progressive worsening without history of vocal abuse.

During laryngoscopic examination we found she did not have structural alterations, but rather a tumor that involved her entire vocal fold. Slow growth and tumor macroscopic traits indicated it was a benign lesion. The most common benign neoplasms at this age range are papillomas and hemangiomas. Papillomas are caused by HPV (human papilloma virus) infection, which is characterized by laryngeal benign epithelial proliferation. Papillomas are the most frequent pediatric laryngeal tumor. They are vegetative tumors with uneven surfaces, and often relapse after surgical removal. They tend to form respiratory blockages and involve patients younger than the one seen in our study. Besides, they are macroscopically different from GCTs. Hemangiomas rank second in prevalence among children, but they occur much less frequently than papillomas. Most are capillary lesions and in 50% of the cases they are associated to cutaneous hemangiomas. They produce biphasic stridor and dyspnea, whose symptoms are present within the first months of life. These tumors are smooth, red or bluish lesions usually located in the subglottis close to the posterior commissure, and tend to resolve spontaneously within 12–18 months. They are macroscopically similar to GCTs, but their site of onset is different from that of the presented case. Besides, onset usually occurs at a younger age and scarcely ever will these tumors persist asymptomatically until early adolescence.

Other less common lesions are polyps and granulomas. Polyps are usually connected to vocal abuse and are gel-like, smaller structures when compared to GCTs, which are more on the fibrotic side. Laryngeal local trauma is connected to the etiology of granulomas, whether it is physical or chemical. They are similar to GCTs macroscopically, but are also smaller and usually located in the posterior larynx. They are rare in children and do not evolve so slowly.

The laryngoscopic aspect and slow growth of GCTs may mislead physicians into treating this condition carelessly, failing to give it the importance it deserves. GCTs are not pre-malignant tumors, nor do they evolve into malignancy; but they may coexist with carcinoma. When not combined with carcinoma, the diagnosis of malignant manifestations is based on accentuated cell pleomorphism and increased mitotic activity; characterizing it though as a malignant tumor can be a quite difficult task. This fact should be taken into account when planning patient treatment, as an over-conservative approach may allow aggressive tumors to remain undiagnosed. GCT treatment is principally surgical, and the tumor must be removed with broad margins as relapse rates increase when margins are compromised. In the case described in this paper we chose to perform a frontal-lateral laryngectomy to fully remove the tumor, as the endoscopic approach would limit that possibility. Even when margins are free of tumor, patients must be followed regularly for long periods of time as the tumor may relapse.

Another important aspect is the likely multicentric manifestation of GCTs. Patients with tumors localized in the upper airways should undergo bronchoscopy to rule out pulmonary tumor foci.

We may therefore conclude that GCTs are rare neoplasms that must be considered in the differential diagnosis of laryngeal tumors. Early diagnosis combined with careful follow-up are required to increase chances of cure.

## CLOSING REMARKS

Granular cell tumors are a rare diagnostic possibility among the tumors involving the larynx. Symptom and critical sign description must be accompanied by ultrastructural and immunohistochemical analysis so a final diagnosis is reached.

Surgery is the recommended treatment for GCTs. The endoscopic approach should be reserved for small tumors, while the external procedure is adequate for larger neoplasms. Complete removal of the tumor is mandatory, and clinical follow-up is fundamental to control relapsing tumors.

## ACKNOWLEDGEMENT

Our gratitude goes to Professor Dr. Donato Próspero from the Pathology Department at Santa Casa de São Paulo, for enabling the conduction of all pathology and immunohistochemistry tests required for diagnosis and documentation of the presented clinical case.
